# Prevalence and associated factors of pistol grip deformity in Japanese local residents

**DOI:** 10.1038/s41598-021-85521-x

**Published:** 2021-03-16

**Authors:** Takaya Taniguchi, Teiji Harada, Toshiko Iidaka, Hiroshi Hashizume, Wataru Taniguchi, Hiroyuki Oka, Yoshiki Asai, Shigeyuki Muraki, Toru Akune, Kozo Nakamura, Hiroshi Kawaguchi, Munehito Yoshida, Sakae Tanaka, Hiroshi Yamada, Noriko Yoshimura

**Affiliations:** 1 Department of Orthopaedic Surgery, Wakayama Medical University, 811-1 Kimiidera, Wakayama, Wakayama Japan; 2 Department of Preventive Medicine for Locomotive Organ Disorders, 22nd Century Medical and Research Center, The University of Tokyo, Hongo, Bunkyo-ku, Tokyo, Japan; 3 Department of Medical Research and Management for Musculoskeletal Pain, 22nd Century Medical and Research Center, Faculty of Medicine, The University of Tokyo, Hongo, Bunkyo-ku, Tokyo, Japan; 4 Rehabilitation Services Bureau, National Rehabilitation Center for Persons with Disabilities, 4-1 Namiki, Tokorozawa, Saitama Japan; 5 Department of Orthopaedic Surgery, Towa Hospital, Towa, Adachi-ku, Tokyo, Japan; 6 Department of Orthopaedics and Spine, Tokyo Neurological Center, 4-1-17 Toranomon, Minato-ku, Tokyo, Japan; 7 Department of Orthopaedic Surgery, Faculty of Medicine, The University of Tokyo, 7-3-1 Hongo, Bunkyo-ku, Tokyo, Japan

**Keywords:** Medical research, Signs and symptoms

## Abstract

Pistol grip deformity (PGD) may be the main factor in femoroacetabular impingement development. This study aimed to clarify the epidemiological indices and factors related to PGD in Japanese people. This population-based cohort study included 1575 local Japanese residents. PGD, center edge angle, and joint space width were measured radiographically. We investigated the relationship between PGD and spino-pelvic parameters. Factors associated with PGD were examined using multiple logistic regression analysis, with the presence/absence of PGD as an objective variable, and sex, age, body mass index (BMI), and the presence/absence of hip pain or spino-pelvic parameters as explanatory variables. In the entire cohort, 4.9% (10.6% men, 2.1% women) had PGD on at least one side. A trend was observed between PGD and increasing age in both men and women (men: p < 0.0001, women: p = 0.0004). No relationship was observed between PGD and hip pain (risk ratio 1.0 [95% confidence interval 0.97–1.03]). Factors significantly associated with PGD were age, sex, and BMI in the multivariate model. Acquired factors may be related to PGD in Japanese people as the PGD prevalence increased with age and PGD was not significantly associated with hip pain. This study provides new insights into the etiology and clinical significance of PGD.

## Introduction

Patients with primary osteoarthritis of the hip often experience swelling in the lateral part of the femoral head-neck junction. This occurrence is termed pistol grip deformity (PGD)^1,2^. PGD has attracted significant attention since several authors have reported the disease concept of femoroacetabular impingement (FAI)^3–6^. There are three types of hip impingements: cam, pincer, and combined. The cam-type is due to an abnormal morphology of the junction between the femoral head and neck, while the pincer type is due to an excessively deep acetabular floor. PGD is presumably the main factor of cam-type FAI in European countries and the United States^7^. In recent years, many reports have suggested FAI as an etiology of hip pain and primary/idiopathic osteoarthritis^8,9^. PGD is considered a congenital deformity^10^. In addition, several studies have suggested an association between PGD and spino-pelvic parameters, including pelvic incidence^11,12^.

Owing to technical advances in magnetic resonance imaging (MRI) diagnostics and hip arthroscopy, the presence and pathology of FAI have begun to attract attention in Japan, where secondary osteoarthritis of the hip due to acetabular dysplasia is common^13^. However, the epidemiology of PGD remains unclear, and the association between PGD and hip pain or primary osteoarthritis has not been elucidated among the Japanese population. Further, the relationship between PGD and spino-pelvic parameters is unknown in this population.

This study aimed to clarify the prevalence of PGD in different age groups and sexes and elucidate related factors, including hip pain, osteoarthritis, and spino-pelvic parameters, in a Japanese population.

## Results

### Prevalence of pistol grip deformity

Table 1 shows the prevalence of PGD in the overall population and the subgroups of age, classified by sex. In the overall population, the prevalence of PGD was 73/1481 (4.9%). The prevalence of PGD in men (52/491; 10.6%) was significantly higher than that in women (21/990; 2.1%). The risk ratio for men was 4.99 (95% confidence interval (CI) 3.04–8.19), which was higher than that for women. Moreover, the prevalence of PGD significantly increased with age for both sexes (Supplementary Figure S1).

**Table 1:** Prevalence of PGD by sex and five age groups.

	Men	Women
**Age groups (years)**		
≤ 49	4/59 (6.7%)	0/117 (0%)
50–59	0/78 (0%)	0/186 (0%)
60–69	5/130 (3.9%)	5/292 (1.7%)
70–79	21/135 (15.6%)	11/278 (4.0%)
≥ 80	22/89 (24.7%)	0.0 (4.3%)
Total	52/491 (10.6%)	0.0 (2.1%)

The prevalence of PGD tends to increase with age group in both sexes (Cochran-Armitage test; men: p < 0.0001, women: p = 0.0004).

*PGD* pistol grip deformity.

### Comparison of the demographics between the groups with and without pistol grip deformity

The PGD-positive group had a significantly higher proportion of men, older age, and higher body weight and body mass index (BMI) than the PGD-negative group (Table 2).

**Table 2:** Comparison of the demographics between the groups with and without PGD.

	PGD (+)	PGD (−)	p-value
Number of participants (hips)	73 (102)	1408 (2860)	
**Demographics**			
Sex (men vs. women)	**52 vs. 21**	**439 vs. 969**	** < 0.0001**
Age (years)	**75.3 ± 11 (72.6–77.9)**	**64.8 ± 12.8 (64.1–65.5)**	** < 0.0001**
Height (cm)	157.4 ± 9.8 (155.1–159.7)	156.2 ± 9.1 (155.7–156.7)	0.2687
Weight (kg)	**59.5 ± 11 (56.9–62.1)**	**56.3 ± 11.2 (55.7–56.9)**	**0.0172**
BMI (kg/m^2^)	**23.9 ± 3.4 (23.1–24.7)**	**23.0 ± 3.5 (22.8–23.1)**	**0.0231**
Hip pain	2/102 (2.0%)	56/2860 (2.0%)	0.9984
**Hip parameters**			
CE angle (° )	**31.3 ± 6.3 (30.1–32.6)**	**28.9 ± 6.4 (28.7–29.2)**	** < 0.0001**
mJSW (mm)	4.0 ± 0.9 (3.8–4.2)	3.9 ± 0.8 (3.9–3.9)	0.5042
**Spinopelvic parameters**			
LL (°)	**40.8 ± 18 (36.5–44.9)**	**45.7 ± 13.3 (45.0–46.4)**	**0.0023**
SS (°)	**28.8 ± 11 (26.3–31.2)**	**31.9 ± 9.0 (31.4–32.4)**	**0.0042**
PT (°)	**21.9 ± 11 (19.4–24.5)**	**18.0 ± 9.1 (17.5–18.5)**	**0.0003**
PI (°)	50.7 ± 12.5 (47.8–53.6)	49.9 ± 10.6 (49.4–50.5)	0.5287

Prevalence of hip pain and hip parameters are calculated from 2962 hips.

*PGD* pistol grip deformity, *BMI* body mass index, *CE angle* center–edge angle, *mJSW* mean joint space width, *LL* lumbar lordosis, *SS* sacral slope, *PT* pelvic tilt, *PI* pelvic incidence.

Data are means ± standard deviation (95% confidence interval). Bold values indicate statistically significant differences between the two groups.

### Association of pistol grip deformity with hip pain

Among the 102 PGD-positive hips, only two participants (2.0%) experienced hip pain on the affected side. No significant association was observed between PGD and hip pain (risk ratio 1.0 [95% CI 0.97–1.03]) (Table 2).

### Association of pistol grip deformity with hip parameters

The PGD-positive group had a significantly larger center edge (CE) angle than the PGD-negative group (Table 2). However, multiple logistic regression analysis, including sex, age, BMI, CE angle, and medial joint space width (mJSW) as explanatory variables, revealed that the presence of PGD was not significantly associated with CE or mJSW. Male sex, age, and BMI were significantly associated with the presence of PGD in this model (Table 3).

**Table 3:** Association of PGD with hip parameters (multivariate analysis).

	Reference	Odds ratio	95% CI	p-value
Sex	**Men vs. women**	**4.54**	**2.85–7.25**	** < 0.0001**
Age	** + 1 year**	**1.09**	**1.07–1.12**	** < 0.0001**
BMI	** + 1 kg/m**^**2**^	**1.12**	**1.06–1.19**	**0.0002**
CE angle	+ 1°	1.03	0.99–1.07	0.1252
mJSW	+ 1 mm	0.91	0.68–1.22	0.537

Odds ratios are calculated by multiple logistic regression analysis on 2962 hips.

*PGD* pistol grip deformity, *95% CI* 95% confidence interval, *BMI* body mass index, *CE angle* center–edge angle, *mJSW* mean joint space width.

Bold values indicate a statistically significant association with PGD.

### Association of pistol grip deformity with spino-pelvic parameters

Regarding the comparison of spino-pelvic parameters, the lumbar lordosis (LL), sacral slope (SS), and pelvic tilt (PT) were found to be significantly different between the groups with and without PGD. Pelvic incidence (PI) was not significantly different between the two groups (Table 3). Subsequently, we conducted a multiple logistic regression analysis wherein the LL, PT, and PI served as explanatory variables, and age, sex, and BMI served as adjusted variables. SS was not included in this model to avoid multicollinearity because we used the algorithm “PI = PT + SS.” Moreover, the propensity score for the objective variable was calculated from the adjusted variables and treated as one variable to avoid overfitting. The presence of PGD was not significantly associated with any of the spino-pelvic parameters; however, PT demonstrated a positive odds ratio (95% CI 1.00–1.10) (Table 4).

**Table 4:** Association of PGD with spino-pelvic parameters (multivariate analysis).

	Reference	Odds ratio	95% CI	p-value
LL	+ 1°	1.00	0.97–1.03	0.7812
PT	+ 1°	1.04	**1.00–1.10**	0.0746
PI	+ 1°	0.99	0.95–1.04	0.8963

Bold values indicate a positive association with PGD.

Odds ratios are calculated by multiple logistic regression analysis after adjustment by age, sex and body mass index on 1481 participants. In creating the model, the propensity score for the objective variable is calculated from the adjusted variables and treated as one variable.

*PGD* pistol grip deformity, *95% CI* 95% confidence interval, *LL* lumbar lordosis, *PT* pelvic tilt, *PI* pelvic incidence.

## Discussion

The most notable result of this study was that the prevalence of PGD increased with age in both men and women. In addition, PGD was significantly associated with weight and BMI. These findings may suggest that the etiology of PGD involves acquired factors in addition to congenital morphological anomalies of the femoral neck, at least in the Japanese population.

In this study, the overall prevalence of PGD was 4.9% (10.6% in men and 2.1% in women). To the best of our knowledge, this is the first study to successfully elucidate the epidemiological indices of PGD and the related factors in a large (approximately 1500) Asian population. A previous study by Goodman et al. examined 2665 cadavers and found 215 cadavers with PGD (8%)^14^. In another study by Doherty et al., the overall prevalence of PGD was 3.61% (6.37% in men and 0.39% in women)^15^. That study included people who had been in the hospital for intravenous urography, with no radiographic hip osteoarthritis. Consistent with their findings, our results suggested that the prevalence of PGD in the Asian participants (Japanese) was relatively lower than that in Europe and the United States. Similar to our results, several previous studies have reported that PGD is more common in men than in women^16,17^. Goodman et al. have reported 122 cadavers with a bilateral PGD incidence of 56.7%^14^, which is considerably high, similar to that in our study (29/71; 40.8%).

Interestingly, the most notable result of this study was that the prevalence of PGD increased with age in both men and women. No past studies have reported a correlation between the prevalence of PGD and age; rather, it has been reported that the prevalence of PGD in men tended to reduce with age^15^. Allen et al. have examined 113 cases with cam-type FAI and reported that a deformity of the head-neck junction could be congenital^18^. However, our findings suggest that the etiology of PGD involves acquired factors in addition to congenital morphological anomalies of the femoral neck in the Japanese population. Only racial differences could cause variations in the correlation of age with PGD. However, regarding the relationship between PGD and spino-pelvic parameters, only PT showed a possible correlation with PGD in our study. An increase in PT indicates pelvic retroversion with age. Thus, instability due to decreased coverage of the antero-lateral femoral head, resulting from pelvic retroversion between the acetabulum and femoral head, might result in PGD as a physiological response.

Another notable result of this study was that the presence of PGD alone was not correlated with either hip pain or joint space narrowing. Based on previous studies from Western countries, PGD is considered susceptible to impingement between the anterior femoral neck and the adjacent acetabulum, which is a common cause of hip pain, labral tears, and osteoarthritis^5,15,18–20^. The difference between our study results and those of previous reports is probably due to the differences in hip joint morphology between Japanese and Western people. Nakamura et al. have evaluated 254 normal hip joints on radiographs and reported that the acetabular roofs of Japanese people are shallower than those of Europeans and Americans^21^. Moreover, Takeyama et al. reported that the anteroposterior diameter of the femoral neck is smaller in the Japanese than in Europeans and Americans, and an impingement between the anterior femoral neck and the adjacent acetabulum is less likely in this population^22^. Our findings suggest that the pathological significance of PGD differs between Japan and Western countries. We should consider that this study excluded patients who had hip osteoarthritis (Kellgren-Lawrence grade ≥ 3) and those who had undergone total hip arthroplasty or bilateral hip arthroplasty. Symptoms might not necessarily appear until PGD progresses to hip osteoarthritis. However, historically, Takeyama et al. retrospectively investigated 946 hip joints of patients who underwent hip surgery and reported the prevalence of characteristic findings of FAI (≒PGD) to be 0.6% in Japan^22^. The actual prevalence of FAI and associated factors, such as hip pain, remain unclear because only a few epidemiological studies on PGD have been conducted in Japan.

This study had several limitations. First, causal relationships between the evaluated variables were not determined because of the cross-sectional study design. Follow-up with a longitudinal study is needed in this cohort. Second, a selection bias might have existed. Among the participants, the proportion of elderly individuals was high because the Research on Osteoarthritis/Osteoporosis Against Disability (ROAD) study was instituted to elucidate the epidemiology of degenerative diseases of locomotive organs. Moreover, regional selection bias should be considered because the subjects (voluntary participants) were recruited from only two regions. However, Yoshimura et al. reported that the participants of the ROAD study are considered representative of the Japanese population^23^. Thus, these findings may not be generalizable to other populations. Applying the results of this study to different races or countries with different lifestyles would require careful judgment. In the future, we will prospectively investigate the percentage of PGD-positive patients who gradually develop osteoarthritis of the hip and factors affecting the transition from PGD to osteoarthritis of the hip using subsequent ROAD study data.

This study clarified the epidemiological index of PGD and related factors in a general population of Japan. PGD has been considered a congenital morphological variation. However, the results of this study indicate that several acquired factors can be associated with PGD, at least in Japanese people, because the prevalence of PGD increases with age. Moreover, PGD was not significantly associated with hip pain. Although the study is limited by its cross-sectional design and potential selection bias, we believe our results provide new insights into the etiology and clinical significance of PGD.

## Materials and methods

### Participants

**Figure 1:**
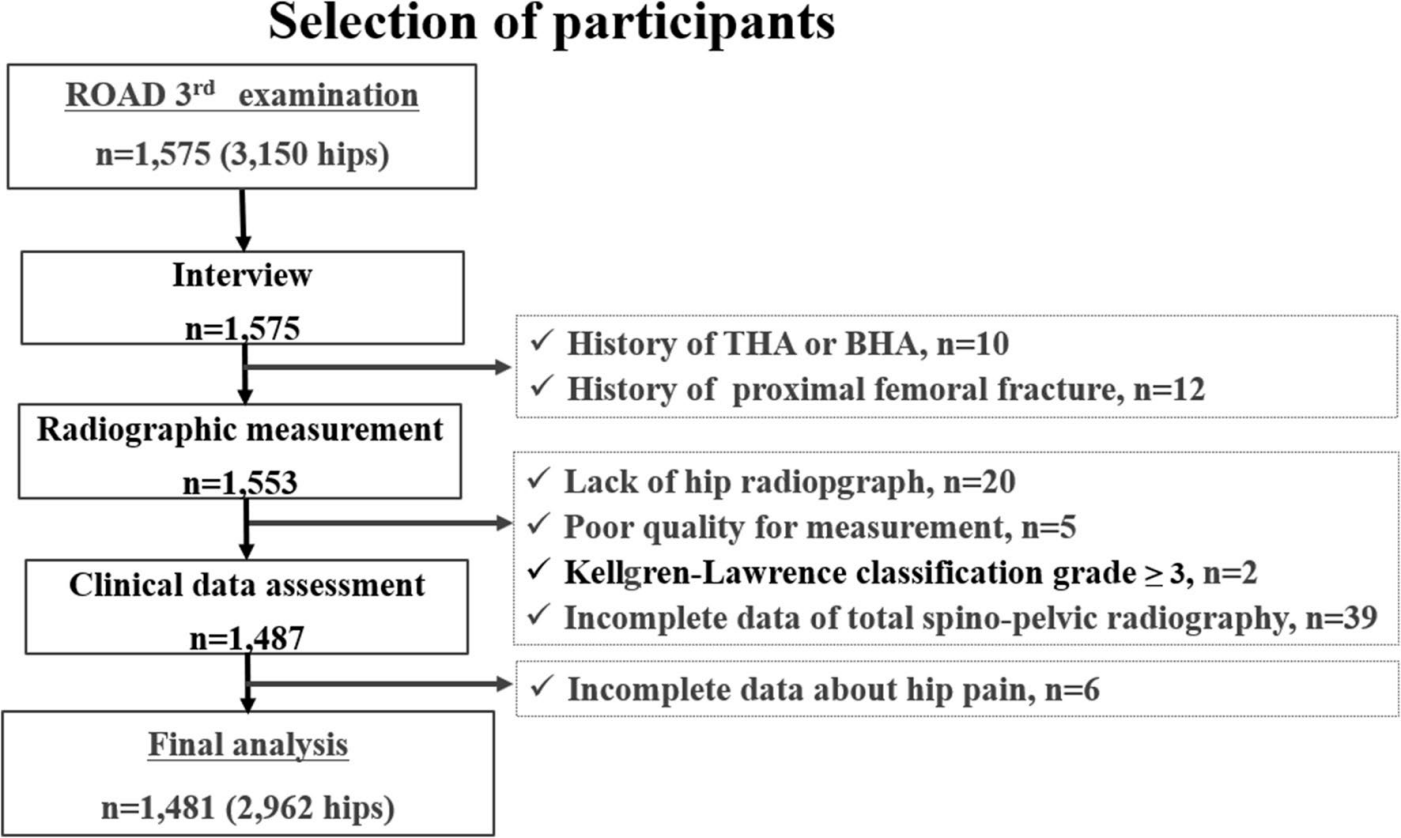
Flow diagram depicting the study enrollment strategy. Participants in the study are recruited from the residents who participated in the 2012 and 2013 examinations for clinical evaluation, as a part of the Research on Osteoarthritis/Osteoporosis Against Disability (ROAD) study. THA, total hip arthroplasty; BHA, bipolar hip arthroplasty.

The ROAD study is a nationwide prospective study of bone and joint diseases comprising population-based cohorts from several communities in Japan. The representative bone and joint diseases of the ROAD study are osteoarthritis and osteoporosis. A detailed profile of the ROAD cohort has been previously reported^23–28^. Briefly, the participants in this study included those recruited during the 3rd examination of the ROAD study, which was conducted from October 2012 to December 2013. In addition to the previous participants, inhabitants of the mountainous area in Hidakagawa (with a population of 11,300 people/330 km^2^) and coastal areas in Taiji (3,500 people/6 km^2^) in the Wakayama prefecture (987,500 people/4725 km^2^) of Japan who were willing to participate in the ROAD survey were also included during this 3rd examination. Overall, 1575 individuals (513 men, 1062 women) participated in the 3rd examination of the ROAD study. Of these participants, 20 (1.3%) did not undergo plain radiography, 7 (0.4%) could not read, 12 (0.8%) had proximal femur fracture, 10 (0.6%) had undergone total hip arthroplasty or bilateral hip arthroplasty, and 2 (0.1%) had hip osteoarthritis (Kellgren-Lawrence grade ≥ 3). In total, 94 individuals, including 43 participants (2.7%) with incomplete spine or hip data, were excluded. The remaining 2962 hips of 1481 participants (94.0%) aged 19–94 years (mean 65.3 years) were included in this study (Fig. 1). The characteristics of the participants are shown in Table 5.

**Table 5:** Characteristics of the participants.

	Total	Men	Women
Number of participants (hips)	1481 (2962)	491 (982)	990 (1980)
**Demographics**			
Age (years)	65.3 ± 13.0 (64.7–66.0)	66.2 ± 13.8 (65.0–67.4)	64.9 ± 12.5 (64.1–65.7)
Height (cm)	156.3 ± 9.1 (155.8–156.7)	164.8 ± 7.2 (164.2–165.5)	152.0 ± 6.6 (151.6–152.4)
Weight (kg)	56.4 ± 11.3 (55.8–57.0)	64.6 ± 11.4 (63.6- 65.6)	52.4 ± 8.7 (51.8–52.9)
BMI (kg/m^2^)	23 ± 3.5 (22.8–23.2)	23.7 ± 3.5 (23.4–24.0)	22.7 ± 3.5 (22.4–22.9)
**Hip parameters**			
CE Angle (° )	29.0 ± 6.4 (28.8–29.3)	29.9 ± 6.0 (29.6–30.3)	28.6 ± 6.5 (28.3–28.9)
mJSW (mm)	3.9 ± 0.8 (3.9–3.9)	4.2 ± 0.8 (4.1–4.2)	3.8 ± 0.7 (3.7–3.8)
**Spinopelvic parameters**			
LL (°)	45.4 ± 13.7 (44.7–46.1)	44.1 ± 12.9 (43.0–45.3)	46.1 ± 14.0 (45.2–47.0)
SS (°)	31.8 ± 9.1 (31.3–32.2)	31.5 ± 8.7 (30.8–32.3)	31.9 ± 9.4 (31.3–32.5)
PT (°)	18.2 ± 9.2 (17.7–18.7)	15.6 ± 7.8 (14.9–16.3)	19.5 ± 9.6 (18.9–20.0)
PI (°)	49.9 ± 10.7 (49.4–50.5)	47.2 ± 9.9 (46.3–48.0)	51.3 ± 10.8 (50.7–52.0)

Data are means ± standard deviation (95% confidence interval of the mean).

Hip parameters were calculated from 2962 hips.

*BMI* body mass index, *LL* lumbar lordosis, *S* sacral slope, *PT* pelvic tilt, *PI* pelvic incidence.

Radiographs of the pelvis, including the hip joints and lumbar spine, were available for the 1481 participants (491 men, 990 women).

The participants completed an interviewer-administered questionnaire, which included questions on family history, medical history, and previous history of hip injury. Anthropometric measurements included height and weight, from which the BMI (weight [kg]/height [m^2^]) was calculated. Furthermore, all participants were interviewed by well-experienced orthopedists regarding pain in both hips. The orthopedists asked, “Have you experienced right hip pain on most days in the past month, in addition to now?” and “Have you experienced left hip pain on most days in the past month, in addition to now?” Participants who answered “yes” were considered to have hip pain.

### Radiographic evaluation

**Figure 2:**
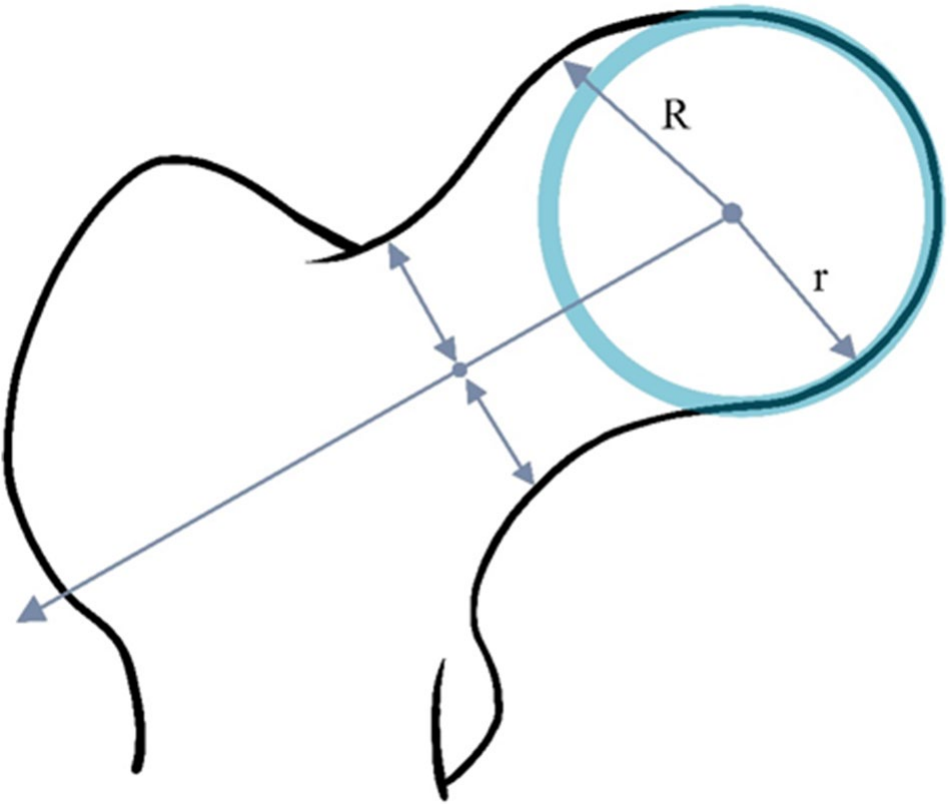
Schema of the definition of a PGD-positive case. The overhang is measured by drawing a circle around the femoral head on the anteroposterior view. Even a small overhang of the femoral head (R > r) is considered a PGD-positive case. PGD, pistol grip deformity.

All participants underwent radiographic examination of both hips using the anteroposterior view with weight-bearing and the feet internally rotated. Fluoroscopic guidance with a horizontal anteroposterior X-ray beam was used to properly visualize the joint space. The hip radiographs were read by three well-experienced orthopedic surgeons who were blinded to the participant's clinical status. The following parameters were measured: existence of PGD^20,29^, CE angle, and mJSW^30^. Based on past reports by Tannast et al.^31^ and Fukushima et al.^32^, we defined PGD as a prominent lateral offset of the femoral head-neck junction. By drawing a circle around the femoral head on the anteroposterior view, the overhang was measured. Instances wherein the femoral head overhang was small were similarly considered PGD-positive cases (Fig. 2).

To evaluate the intra-observer variability of the hip parameters, 50 randomly selected radiographs of the hip were analyzed by the same observer > 1 month after the first reading. Additionally, 50 other radiographs were analyzed by the three orthopedic surgeons using the same atlas for interobserver variability. The intra-class and inter-class correlation coefficients for the CE angle were 0.94 and 0.85, respectively, and 0.97 and 0.97, respectively, for mJSW. The interobserver variability for the center of the femoral head was confirmed by kappa analysis to be sufficient for assessment (κ = 1).

**Figure 3:**
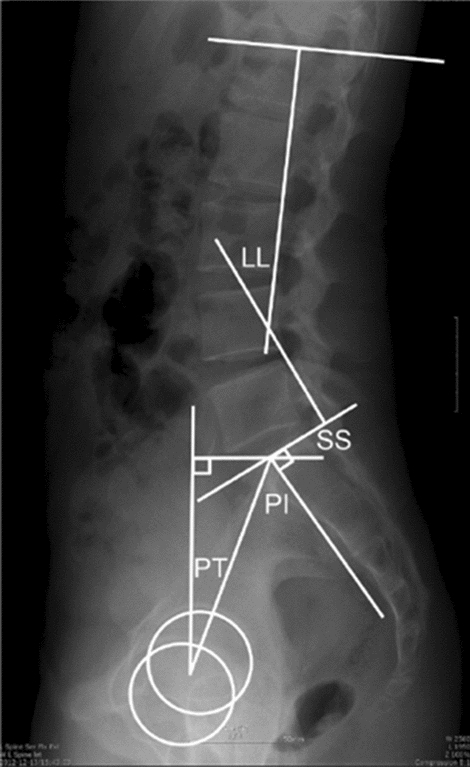
Schema of the measuring methods for each spino-pelvic parameter on a standing radiograph of the whole spine and pelvis. LL, lumbar lordosis; SS, sacral slope; PI, pelvic incidence; PT, pelvic tilt.

Moreover, for spino-pelvic alignment parameters, standing lateral radiographs of the whole spine and pelvis were taken. Each radiograph was aligned such that the edge of the film was the reference for vertical alignment. On the standing lateral radiographs, the following parameters were measured: LL (the Cobb angle from the upper endplate of L1 to the lower endplate of S1), SS (the angle between the tangent line to the superior endplate of S1 and the horizontal plane), PT (the angle between the line connecting the midpoint of the sacral plate to the axes of the femoral heads and the vertical axis), and PI (the angle between the line perpendicular to the sacral plate at its midpoint and the line connecting this point to the axes of the femoral heads) (Fig. 3)^33,34^.

### Statistical analysis

Statistical analyses were performed using JMP (version 14; SAS Institute Inc., Cary, NC). Continuous variables are expressed as the mean ± standard deviation (SD) and 95% CIs. The participants were classified into five age groups based on birth-year decade: (1) less than 50 years, (2) 50–59 years, (3) 60–69 years, (4) 70–79 years, and (5) 80 years and older. The Cochran-Armitage test was conducted to determine the trend of PGD prevalence among the five age groups. Differences in age, height, weight, BMI, hip parameters, and spino-pelvic parameters were examined between men and women or between the PGD-positive and PGD-negative groups using a non-paired t-test. A chi-square test was conducted to compare the prevalence of PGD between men and women or between the groups (with versus without hip pain). The association of the variables, including age, BMI, sex, spino-pelvic parameters, and hip parameters, with the presence/absence of PGD was examined via multiple logistic regression analysis. Risk ratios or odds ratios are provided with 95% CIs.

### Ethics declarations

All participants provided written informed consent for their participation and for the publication of the study in print and in electronic form.

The study was conducted with the approval of the appropriate ethics committees. The establishment of a cohort related to this study has been approved by the Research Ethics Review Committee of the University of Tokyo (No. 1326). The procedures followed were in accordance with the ethical standards of the responsible committee on human experimentation (institutional and national) and with the Helsinki Declaration of 1975, as revised in 2000.

## Supplementary Information

Supplementary Figure Legend.

Supplementary Figure S1.

## Data Availability

All data generated or analyzed during this study are available from the corresponding author upon reasonable request.
