# Supplementary material for: Prevalence and associated factors of pistol grip deformity in Japanese local residents
Source: Sci Rep. 2021 Mar 16;11:6025. doi: 10.1038/s41598-021-85521-x (PMC7966377; doi:10.1038/s41598-021-85521-x)
Supplement: Supplementary file 1 — Supplementary Figure Legend. [file 41598_2021_85521_MOESM1_ESM.docx]

**Supplementary information**

Supplementary Figure S1. Prevalence (percentage) of subjects with PGD (+) in each age group（<50, 50-59, 60-69, 70-79, >80）. The prevalence of PGD significantly increases with age in both sexes using the Cochran-Armitage trend-test.
