# Supplementary figures and images for: Prevalence and associated factors of pistol grip deformity in Japanese local residents
Source: Sci Rep. 2021 Mar 16;11:6025. doi: 10.1038/s41598-021-85521-x (PMC7966377; doi:10.1038/s41598-021-85521-x)

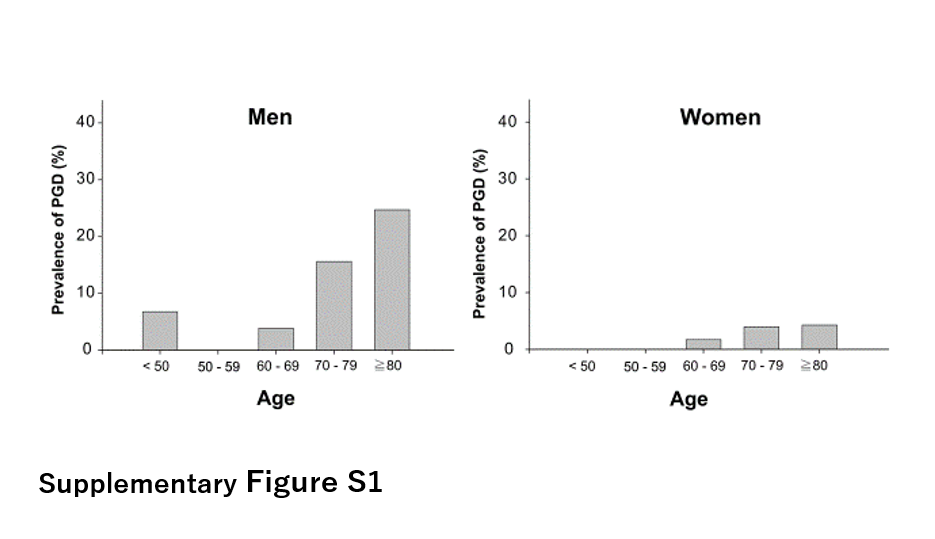

Supplement: Supplementary file 2 — Supplementary Figure S1. [file 41598_2021_85521_MOESM2_ESM.tif]
